# Synthesis and Characterization of a Lignin-Styrene-Butyl Acrylate Based Composite

**DOI:** 10.3390/polym11061080

**Published:** 2019-06-25

**Authors:** Daniel López Serna, Perla Elizondo Martínez, Miguel Ángel Reyes González, Antonio Alberto Zaldívar Cadena, Erasto Armando Zaragoza Contreras, María Guadalupe Sánchez Anguiano

**Affiliations:** 1 Universidad Autónoma de Nuevo León, Facultad de Ciencias Químicas, Av. Universidad S/N, Cd. Universitaria, 66455 San Nicolás de los Garza, N.L, México; dlslqi@gmail.com (D.L.S.); perlaelizondomx1@gmail.com (P.E.M.); miguelangel138@hotmail.com (M.Á.R.G.); 2 Universidad Autónoma de Nuevo León, Facultad de Ingeniería Civil, Av. Universidad S/N, Cd. Universitaria, 66455 San Nicolás de los Garza, N.L, México; azaldiva70@gmail.com; 3 Centro de Investigación en Materiales Avanzados, S.C. Miguel de Cervantes No. 120, Complejo Industrial Chihuahua, 31136, Chihuahua, Chih. México; armando.zaragoza@cimav.edu.mx

**Keywords:** lignin, polymer composites, thermal properties, hardness, contact angle

## Abstract

In recent years, the pursuit of new polymer materials based on renewable raw materials has been intensified with the aim of reusing waste materials in sustainable processes. The synthesis of a lignin, styrene, and butyl acrylate based composite was carried out by a mass polymerization process. A series of four composites were prepared by varying the amount of lignin in 5, 10, 15, and 20 wt.% keeping the content of butyl acrylate constant (14 wt.%). FTIR and SEM revealed that the –OH functional groups of lignin reacted with styrene, which was observed by the incorporation of lignin in the copolymer. Additionally, DSC analysis showed that the increment in lignin loading in the composite had a positive influence on thermal stability. Likewise, Shore D hardness assays exhibited an increase from 25 to 69 when 5 and 20 wt.% lignin was used respectively. In this same sense, the contact angle (water) measurement showed that the LEBA15 and LEBA20 composites presented hydrophobic properties (whit contact angle above 90°) despite having the highest amount of lignin, demonstrating that the interaction of the polymer chains with the –OH groups of lignin was the main mechanism in the composites interaction.

## 1. Introduction

Lignin, in conjunction with cellulose, is one of the most abundant biopolymers in nature. Lignin is obtained as a by-product from the wood pulp during the paper fabrication [1,2,3]. Due to its chemical structure, based in coniferyl, coumaryl, and sinapyl monomers randomly distributed and crosslinked, lignin has limited use in industrial scale processes [4,5]. Lignin is a natural polymer based in coniferyl, coumaryl, and sinapyl monomers, randomly distributed and crosslinked. It is an amorphous material and hydrophobic branched which has recently been used for industrial applications, in a variety of alternatives [6,7].

During the last decades, a large number of studies have been presented on the potential use of lignin at the industrial level for the production of various materials, mainly as binder and dispersant [8,9], although lignin is a large source of aromatic compounds. The research work at the laboratory level aims to use it for the development of high-value polymers [10,11,12,13,14] or for the production of biofuel; in both cases, the purpose is to reduce the emission of volatile organic compounds (VOC) and greenhouse gases, as CO_2_ [15,16,17,18,19]. Kasko et al. presented a review on the strategies to use lignin [20], where most of the research focuses on the methods developed for the synthesis of polymers from this biopolymer and its derivatives [20,21,22], giving a great value to the environmental benefit that entails the use of lignin as a raw material [23,24]. Other studies have focused on the application of lignin as a nanofiller [25] or in storage energy [26].

On the other hand, the study of the synthesis of compounds and nanocomposites through the grafting of lignin with acrylic monomers has been deepened [27,28], which have served as the basis for studying the compatibility and reinforcement effect of lignin blend whit different monomers [29], as well as the analysis of the compatibility of mixtures of lignin with epoxy resins [30,31]. The research on the preparation of lignin with various polymeric materials with different applications has been studied from the viewpoint of matrix reinforcement with fibers or particles of different morphology [32,33,34]. The nature of the matrix varies from a synthetic polymer to a natural one, depending on the application [35,36,37]. It has been found that both the polymeric matrix and the reinforcement play an important role in the determination of the physicochemical properties of the composite materials in general [16,38,39,40,41]. Fatehi, et al. reported the modification of a polymer of lignin-g-styrene and its application for wastewater treatment and water purification, by introducing a sulfonate group in the polymer matrix that modifies the anionic charge [42].

**Scheme 1:**
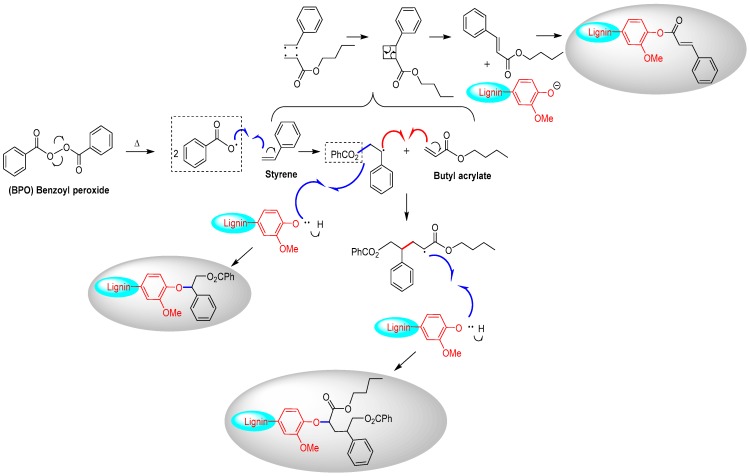
Reaction mechanism for the chemical interaction between lignin and styrene-butyl acrylate copolymer.

Under this context, we present the synthesis of a new compound based on lignin, styrene, and butyl acrylate, obtained by a bulk free radical polymerization. In the mechanism suggested in Scheme 1, styrene is added to the –OH groups of lignin through a free radical reaction. To understand the relationship between the lignin amount and the composite properties, the thermal properties, the contact angle, and the copolymer molecular weight, with and without lignin, were studied. The purpose was to deepen in the composite behavior to be able to visualize some potential applications; for example, as an adsorbent material to remove water pollutants, for synthetic wood, or for the absorption of hydrocarbons spills in water.

## 2. Experimental

### 2.1. Materials

Lignin Kraft (98%, Sigma Aldrich, St. Louis, MO, USA) was dried in an oven for 24 h at 110 °C to remove moisture, the main characteristics of lignin are Mw 28,000 g mol^−1^ and Mn 5000 g mol^−1^. Styrene monomer (98% DEQ, México), butyl acrylate (97% Sigma Aldrich), and benzoyl peroxide (99% Sigma Aldrich). All reagents were used without further purification.

### 2.2. Composite Synthesis

The composite synthesis was performed by bulk free radical polymerization, mixing the defined amount of styrene (S), butyl acrylate (BA) and lignin, keeping the reagents total amount of 4.0 g. Benzoyl peroxide (C_14_H_10_O_4_) was fed at 1 wt.% with respect to the total amount of the reaction mixture. A total of five experiments was performed. Table 1 shows the relationship between lignin and styrene used. The polymerization time for all the experiments was 2 h, at 90 °C, and maintaining a stirring speed of 1200 rpm. At the end of the established time, the materials were cooled rapidly in an ice bath (quenching). The products resulting from the experiments were identified as EBA for the material without lignin and LEBA for the materials containing lignin.

### 2.3. Residual Styrene Analysis by HPLC

To determine the reaction time effect on the incorporation of styrene in the composites, the amount of the residual monomer was analyzed. From the experiment LEBA15, 4.0 g of sample was taken at 5, 10, 15, 30, 45, 60, 75, 90, 135, and 180 min of reaction, the sample was dispersed in methanol under stirring; then the composite was separated from the composite via centrifugation and the unreacted styrene was dissolved in methanol. Finally, the resulted solution was analyzed using a high-resolution liquid chromatograph (HPLC) (YL9100, Young Lin, Anyang, Korea), which has a UV detector (YL9160PDA, Young Lin), a column of 30 cm × 4 mm × 5 μm (SUPELCOSIL LC-18-DB, SUPELCO, Sigma Aldrich), and as the mobile phase a methanol:water (65:35) solution. The samples were run at a flow rate of 1 mL min^−1^ with a volume of 25 μL of injection.

### 2.4. Gel Permeation Chromatography Analysis

In order to study the effect of the nitrogen atmosphere in the polymerization process, the synthesis of the copolymer EBA under nitrogen and air was achieved. The obtained copolymers were analyzed by gel permeation chromatography (GPC) (YL900GPC, Young Lin) using HPLC grade tetrahydrofuran as the mobile phase. The equipment has an autosampler (YL9150), a 5 μm column of 300 × 7.5 mm (MIXED-C PLgel), and a refractive index detector (YL9170). The analyses were determined under the following conditions: Flow rate of 1.0 mL min-1 for 15 min, furnace temperature in the column of 25 °C, and injection volume of 25 μL.

### 2.5. Infrared Spectroscopy Analysis

The composite functional groups were identified using an infrared spectrometer (FTIR380, Nicolet, Thermo Fisher, Waltham, MA, USA) using the KBr pellet method, and scan of 4000 to 650 cm^−1^ with a resolution of 4 cm^−1^, and 32 scans were performed per sample.

### 2.6. Differential Scanning Calorimetry Analysis

The composite thermal behavior was determined by using a differential scanning calorimeter (DSC 8000, Perkin–Elmer, Waltham, MA, USA). The measurements were carried out with 10 mg of sample, in the range of 20 to 350 °C, at a heating rate of 10 °C min-1, under nitrogen atmosphere.

### 2.7. X-ray Diffraction Analysis

The room temperature XRD was performed in a powder X-ray diffractometer (EMPYREAN, PANalytical, Malvern, UK) using Cu-Kα1 wavelength of 1.5406 Å, 40 KV, and 40 mA. The measurement was made within the 2-theta scale from 5° to 45° with a step size of 0.025°.

### 2.8. Nuclear Magnetic Resonance Analysis

The composite structure was also analyzed by proton nuclear magnetic resonance (^1^H-NMR) (600 MHz NMR, Varian, Palo Alto, CA, USA). The measurements were made at room temperature using DMSO-d6 as the solvent.

### 2.9. Morphological Analysis

The morphology was determined in a scanning electron microscope (SEM) model JSM-6510LV brand JEOL (Tokyo, Japan) with an acceleration voltage of 20.0 kV and a spot-size of 60 and working distance of 15 mm. The samples previously were coated with gold/palladium by an electrodeposition method.

### 2.10. Determination of Hardness Shore D

The measured of hardness of the composites with a Shore “D” durometer PRECISION Shore Model D, was used in cubic specimens of 2 cm edge material, the measurements were taken at 10 measuring points at each sample, and the mean values and standard deviations were calculated in accordance to ASTM standard D2240.

### 2.11. Contact Angle Determination

The analysis of the hydrophilic nature of the composites, a RameHart Instruments Model 200 contact angle meter (Succasunna, NJ, USA) was used, using deionized water as test fluid, the reported value in this work was the average contact angle of at least three droplets deposited at different positions of the sample surface.

## 3. Results and Discussion

**Figure 1:**
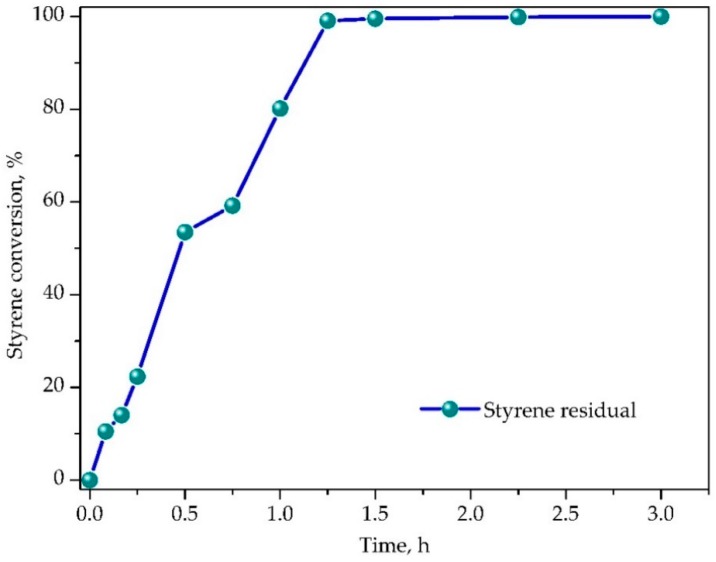
Conversion of styrene versus time in experiment LEBA15.

The butyl acrylate (BA) monomer was included to impart slight flexibility and to prevent obtaining a brittle composite [43]. Figure 1 shows the conversion of styrene in composite LEBA15 as a function of time. As noted, after 1.3 h the residual styrene was less than 0.04%; after this time, the conversion reached a steady state, so the composite synthesis can be stopped. This method of synthesis has advantages over other reports that involve the incorporation of lignin in polymeric matrices, which require the use of solvents or a pre-treatment to lignin [42,44,45] or are necessary longer synthesis time [29,46].

After this time, the conversion reaches a steady state, so the synthesis of the composite can be stopped at 1.5 h of reaction. Short reaction time implies energy savings when the compound is developed at an industrial level.

### 3.1. GPC Analysis

A GPC analysis was performed in the copolymer EBA to determine weight average molar mass (Mw), number average molar mass (Mn) and polydispersity (I = Mw/Mn). In this experiment, the synthesis of EBA was realized under nitrogen (N_2_) and air atmospheres to study the influence of the atmosphere in the composite polymerization. The results are reported in Table 2; as observed, the high polydispersity indicates that there was wide molecular weight distribution. In bulk polymerization, the viscosity of the medium increases gradually along the polymerization time; so, the radical propagation is slow, consequently, the termination of the chains occurs faster than the growth, generating high polydispersity [47,48,49]. A slight increase in the molecular weight and polydispersity was obtained in the copolymer synthesized under N_2_ [48], which implies that the functionality of the initiator is not seriously affected by the presence of oxygen in the air atmosphere, so it is not necessary to use an inert atmosphere.

### 3.2. FTIR Analysis

**Figure 2:**
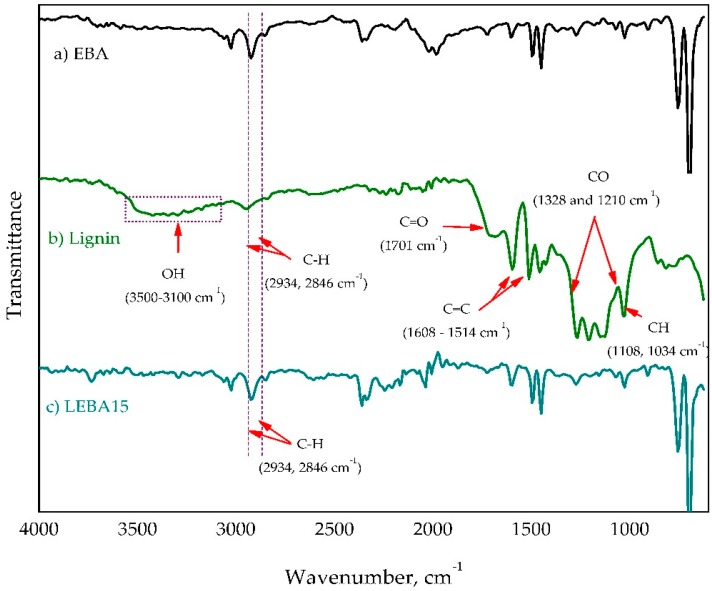
Infrared spectra of (**a**) EBA, (**b**) Lignin, and (**c**) LEBA15.

The FTIR spectra of EBA copolymer, lignin, and LEBA15 composite are shown in Figure 2. For EBA, the main signals are observed at 3100 cm^−1^, corresponding to the stretching vibration of the C–H bond in the aromatic ring of styrene monomer unit; at 2934 and 2846 cm^−1^, assigned to asymmetric and symmetric stretching vibrations, respectively, of the C–H bond of the methylene groups of the polymeric backbone. And at 1731 cm^−1^, the corresponding stretching vibration of the C=O group of the acrylate functionality, which appeared very weak due to the small content in the copolymer (14 wt.%).

The spectrum of lignin exhibited the structural complexity of this biopolymer. The wideband between 3500 and 3100 cm^−1^ was attributed to the stretching vibration of –OH groups; the bands at 2934 and 2846 cm^−1^ were associated with the stretching vibrations of C-H of methylene group, while the band at 1701 cm^−1^ was ascribed to the stretching vibration of C=O of ester groups. The band at 1608 cm^−1^ was attributed to the stretching vibration of C=C bonds in the aromatic ring [50,51,52]. Finally, the signals between 828 and 614 cm^−1^, are due to deformations outside the plane of the aromatic CH bonds, similar to reported by other authors [50,53].

**Figure 3:**
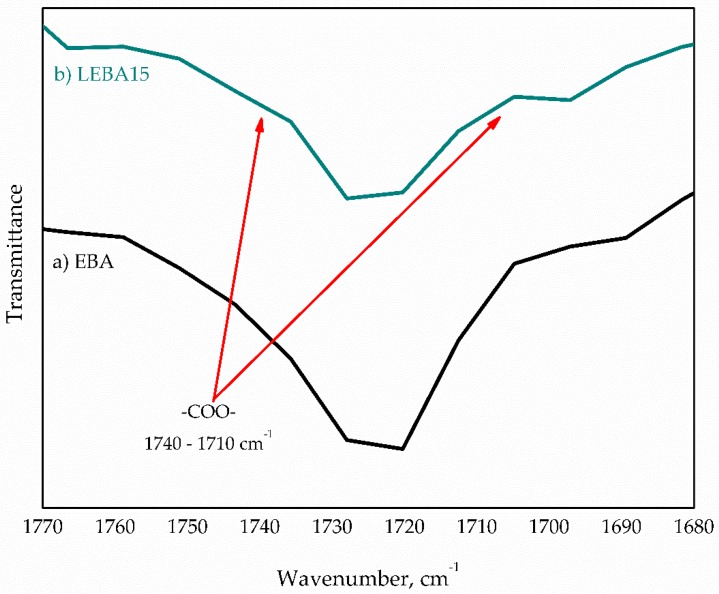
Infrared spectra of (**a**) EBA and (**b**) LEBA15.

In the spectra for LEBA15, the band corresponding of the –OH groups from the lignin not be solved, the intensity decreases because these groups probably react with short chains of monomers, causing the formation of the composite. The formation of bonds in the alcoholic and phenolic groups of lignin has already been reported by Dick et al. [54] in their work about chemical modification and grafting induced by lignin plasma in polyacid lactic acid. In this sense, Fuxiang et al., in various research works, shows that the decrease in the intensity of the band of –OH groups of lignin is the main mechanism of interaction for the incorporation of this with the polymer matrix of interest [29,55]; in this same sense, in this research, a decrease in the intensity of band of the –OH groups of lignin was observed and it is associated with its possible interaction with the acrylate groups of the copolymer (Scheme 1) for the formation of the composites, which is demonstrated by the decrease in the intensity of the band between 1740–1710 cm^−1^ typical of the acrylate for the LEBA15 composite (Figure 3).

### 3.3. DSC Analysis

**Figure 4:**
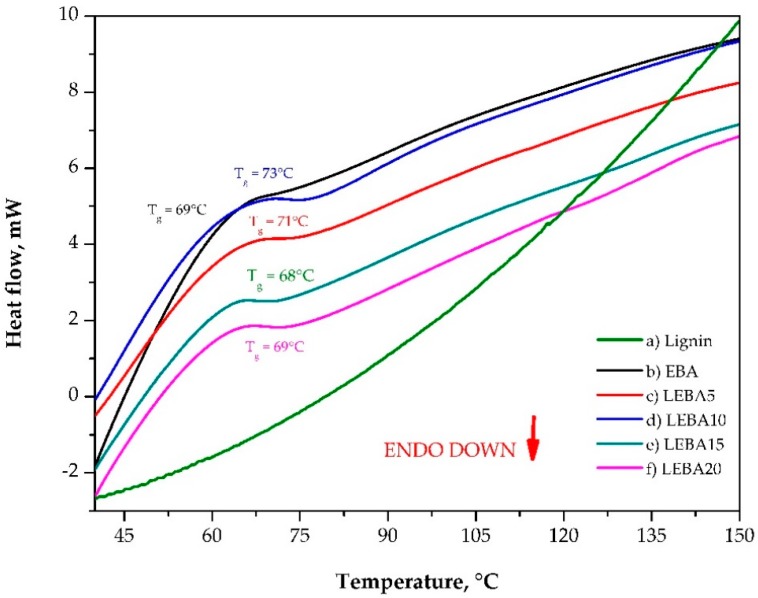
Differential scanning calorimeter (DSC) curves for (**a**) Lignin, (**b**) EBA, (**c**) LEBA5, (**d**) LEBA10, (**e**) LEBA15 and (**f**) LEBA20 composites.

The lignin, copolymer EBA, and LEBA composites were characterized by DSC to determine the glass transition temperature (*T*_g_) (Figure 4). As seen, the *T*_g_ of the composites was slightly lower than the *T*_g_ of the pure copolymer and there seems to be no trend with respect to the lignin content. It is believed that the presence of lignin into the copolymer affected the mobility of the polymer chains [56,57], due to the intermolecular hydrogen bond between hydroxyl functional groups of lignin and the carbonyl groups of the copolymer [58].

However, in the composites LEBA15 and LEBA20 the relationship between S and BA was increased, causing the polymeric chains of these composites to be a little more flexible due to the increase in the BA [59].

### 3.4. X-ray Diffraction Anlysis

**Figure 5:**
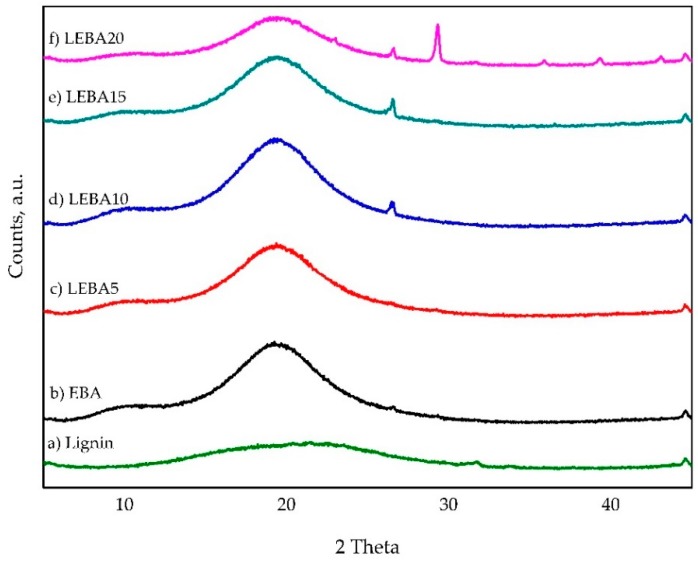
X-ray diffraction patterns for the samples: (**a**) Lignin, (**b**) EBA, (**c**) LEBA5, (**d**) LEBA10, (**e**) LEBA15, and (**f**) LEBA20.

Figure 5 shows the diffraction patterns for the composites, is observed that lignin and EBA did not exhibit any signal of sharp diffraction peaks, indicated that are amorphous materials. At low amount of lignin, the amorphous phase of the composite prevails. However, increasing the amount of lignin appeared peak at 26° on 2 theta for LEBA10 to LEBA20 on the XRD diffraction, because the interaction sites between the lignin and the copolymer are increased, this causes increased probability that the chains fit between gaps of lignin, which makes some structures form ordered aggregation.

However, the composite LEBA20 showed diffraction peaks at higher values of 2 theta, several authors which have already reported this kind of diffractograms to attribute the origin of this additional peak to a scattering signal related to the lateral groups of the same size and almost regularly spaced, this agrees with the fact that LEBA20 has the highest BA/St ratio [60], the main amorphous signal located around 20° results from the contribution of both the lignin and the copolymer chains, the disappearance or decrease in intensity of this signals for the intermediate BA/St ratio indicates a loss of this characteristic length, which may suggest that the grafted chains are rather composed of random copolymers.

### 3.5. ^1^H-NMR Analysis

**Figure 6:**
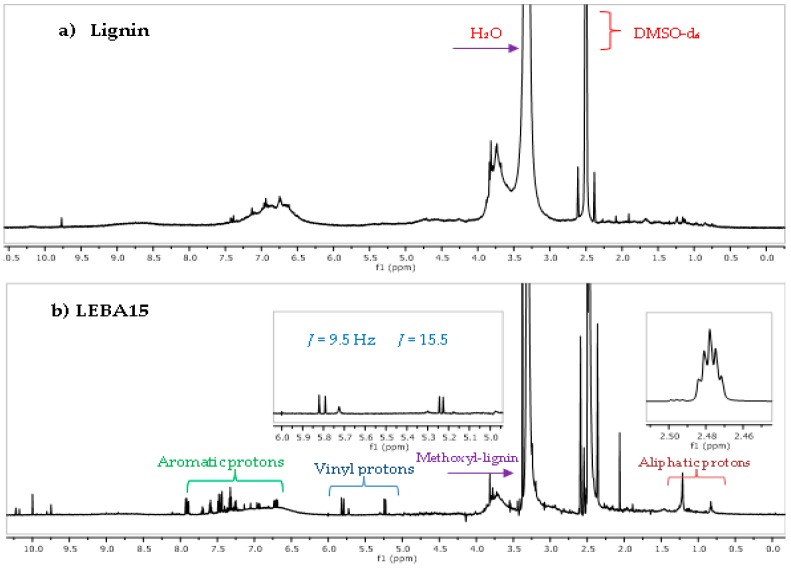
^1^H-NMR spectra (DMSO-d_6_, 25 °C, 400 MHz) for: (**a**) Lignin and (**b**) LEBA15.

By means of the ^1^H NMR characterization of the LEBA 15% composite, a direct comparison was achieved with the ^1^H NMR spectrum of Lignin, where we were able to assign different characteristic signals of the material (Figure 6).

Due to their complex structure, lignins present equally complex spectra; however, they maintain a characteristic pattern. The most common functional groups present in lignins are the methoxy group (Ar–O–CH_3_), various types of ethers (R–O–R, Ar–O–R, Ar–O–Ar), aldehydes (R–H–C=O), phenols (Ar–OH) and aliphatic alcohols (R–OH), among several others. Figure 5 shows the spectrum of the LEBA15 composite. Two signals appear in the spectrum, one in 2.4 ppm and another in 3.35 ppm, the first corresponds to the DMSO signal used as the solvent and the second to the trace of water contained in the DMSO. In the area close to 10 ppm, a small group of signals is observed, which has been related to protons in benzaldehyde groups [61]; a more prominent set of signals between 6 and 9 ppm was attributed to protons of aromatic OH groups in syringyl (S) and guaiacyl (G) units; at 3.75 ppm appears a multiplet attached to the protons of methoxy groups related to S and G units; the signals between 0.6 and 1.25 ppm correspond to the protons of the aliphatic groups. Two doublets are also observed at 5.25 and 5.8 ppm, which are characteristic of protons in unsaturated groups (C=C). Unlike Lignin, which does not have double bonds in its structure. Once it is coupled with styrene and butyl acrylate, it can be observed in 5.25 ppm a doublet with a coupling constant of *J* = 15.5 Hz, in 5.80 another doublet with a coupling constant of *J* = 9.5 Hz which means the presence of two isomers of configuration (E) and (Z), this may be due to the conformational arrangement of the functional groups that lignin presents within its macrocyclic polymer structure. On this type of signals, the literature indicates that they usually occur in arrangements of type β-O-4 [62], remnants of the biopolymerization of monolignols [63].

This set of signals is in agreement with spectra reported in the literature for lignins [61]. With respect to the copolymer, the signals of the repeating units of styrene and butyl acrylate appear to be masked by those of lignin since in most cases the protons are coincident. However, we can mention the multiplet (two double of doubles) at 6.75 ppm observed in polystyrenes and the set of signals between 7.25 and 7.5 ppm attributed to the aromatic ring protons; however, aromatic groups in lignin also occur in this area. As for the repeating units of butyl acrylate, they are not clearly observed, probably due to the low content of this monomer in the composite.

### 3.6. Morphological Analysis

**Figure 7:**
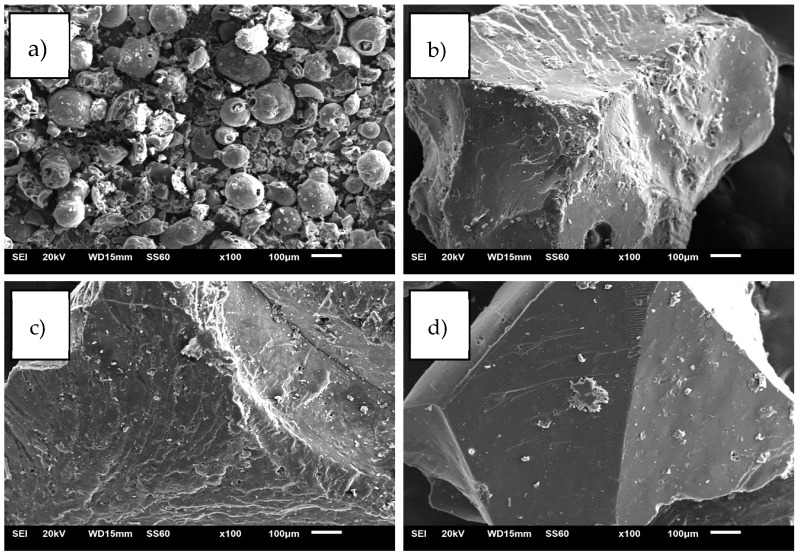
SEM micrographs for samples of (**a**) lignin, (**b**) LEBA5, (**c**) LEBA15, and (**d**) LEBA20.

Figure 7 portrays the morphology for lignin, LEBA5, LEBA15, and LEBA20. The image of the pure lignin (a) showed an irregular distribution of particles in the form of spheres of size varying from 30 to 150 μm.

With the increase of the lignin, the composite surface became smoother (Figure 6b,c,d), suggesting that there was a link between the functional groups of lignin and the copolymer. Similar behavior was reported by Yang et al., they report the preparation of lignin pristine (LP) reinforced polymethyl methacrylate (PMMA) composites, obtained by combining solvent-free radical polymerization, obtaining making the particles totally embedding into the PMMA chain and can be ascribed to excellent bonding effect of LP whit PMMA, this represents increment in the mechanical properties of composites, by not presenting empty spaces between the lignin and the polymeric matrix [64].

### 3.7. Determination of Hardness

**Figure 8:**
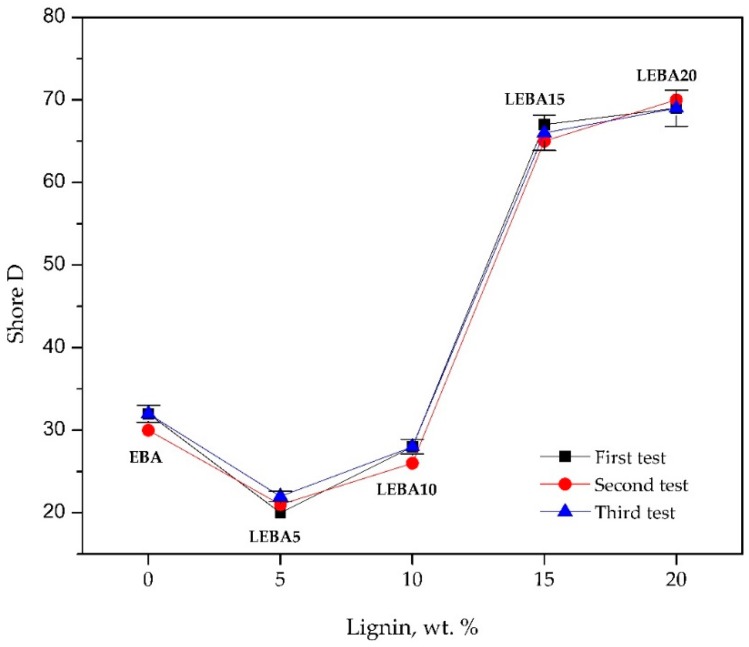
Shore D hardness vs. lignin wt.%**.**

Specimens were prepared from the composites in order to evaluate hardness Shore D, according to ASTM D 2240, the measurements were made by triplicate. As illustrated in Figure 8, the results indicated that the composites with 5 and 10 wt.% lignin had lower hardness than the pure copolymer EBA. However, for the composites with higher contents of lignin an increasing in hardness was observed. Yang et al. present the Shore D hardness values of PMMA material sheets incorporating various contents of LP, with the increase of LP loading from 0 to 4.5 wt.%, the Shore D hardness rise from 75.0 to 79.0 [64].

These results are agreement with the SEM images and DSC supporting the hypothesis that lignin is embedded within the polymer chains forming a material with smooth surface, the incorporation of this biopolymer favored the mechanical properties of LEBA composites, opening the possibility of the application of this material; for example, in the removal of hydrocarbons from water since for this application [43].

### 3.8. Contact Angle

**Figure 9:**
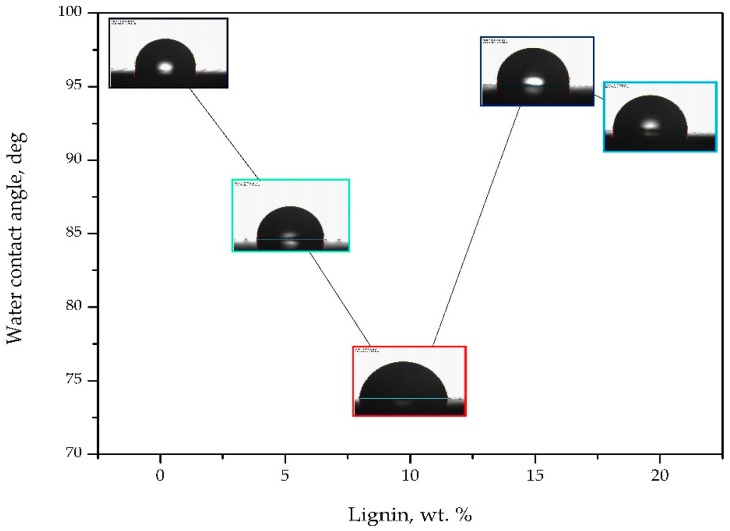
Water contact angle vs. lignin wt.%**.**

Figure 9 shows the results of the determination of the contact angle for the EBA copolymer and the LEBA composites.

**Scheme 2:**
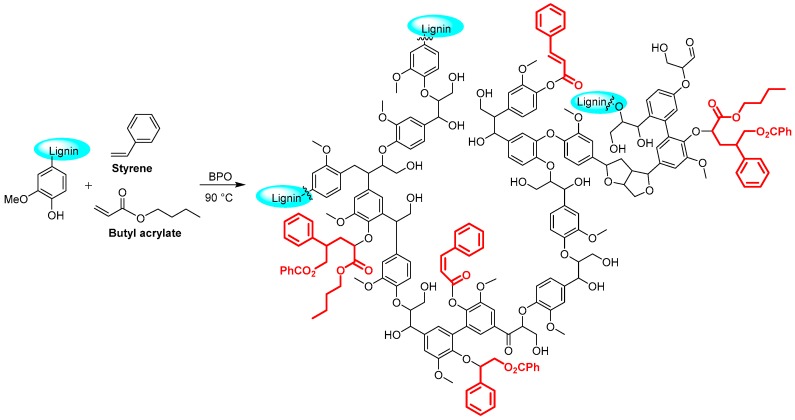
Schematic diagram of the probable links in composites synthetized with lignin and styrene-butyl acrylate.

The contact angle for EBA (97.5°) showed a hydrophobic nature that agrees with that reported in the literature for polymeric materials based on styrene [65,66]; On the other hand, the composites LEBA5 and LEBA10 showed a more hydrophilic character that could be associated with the presence of lignin due to its hydrophilic nature [65]. In contrast, for LEBA15 and LEBA20 the hydrophobic character was accentuated, despite containing higher loads of lignin. This behavior can be attributed to a higher BA/styrene ratio [67]. According to this, the interaction of –OH groups of lignin with the copolymer during free radical polymerization increased (Scheme 2), occupying a higher proportion of this groups, which caused the decrement in the hydrophilic character associated with lignin [65].

In this sense, some research in the synthesis of lignin-based materials, reported that the increase of lignin in the polymer matrix influences the increment of the hydrophilic character (Table 3), because lignin acts as a filler [68]. On the other hand, under the synthesis conditions proposed in this research it was shown that the increase in the amount of lignin (LEBA15 and LEBA20) had no significant effect on the hydrophobic character of the styrene-butyl acrylate system.

## 4. Conclusions

A novel composite was synthetized with lignin and styrene-butyl acrylate copolymer. Under the conditions of the composite formation, the results indicated that the –OH groups of lignin were the main site of bonding with the copolymer chains, this was evidenced by the reduction of the hydroxyl groups signals in infrared spectroscopy. Gel permeation chromatography showed that the atmosphere of nitrogen in the reaction medium did not contribute significantly to the inhibition of the free radicals during copolymerization. In addition, electron microscopy showed that the lignin was incorporated with the copolymer, losing its granular morphology and producing a smooth surface, which was related to FTIR observations. Respecting surface characterization, it was found that lignin loading modified the composites hardness and the hydrophilic-lipophilic character. The more hydrophobic character of LEBA15 and LEBA20 composites supported the assumption of the chemical interaction between the OH groups of lignin and the copolymer chains during free radical polymerization.

According to the properties observed, it is possible to suggest applications for the composites; for example, based on the lipophilic nature, the composites could be used as adsorbent materials for the removal of hydrocarbon spills from water.

## Figures and Tables

**Table 1:** Lignin and styrene quantity present in composites, with butyl acrylate (BA) constant in 14 wt.%.

Composite	Lignin [wt.%]	Styrene [wt.%]	BA/St ratio
EBA	0	86	0.16
LEBA5	5	81	0.17
LEBA10	10	76	0.18
LEBA15	15	71	0.20
LEBA20	20	66	0.21

**Table 2:** Gel permeation chromatography (GPC) results for EBA copolymer with air atmosphere and N_2_.

Atmosphere	Mn	Mw	I
Air	15,404	83,986	5.45
Nitrogen	18,339	87,678	4.78

**Table 3:** Effect of lignin in water contact angle for different polymer matrices.

Polymer	Type lignin	Application	Water Contact Angle, °
Polyurethane (PU) [68]	Lignin waste (5.0 to 20.0 wt.%)	Removal oil in water	123.0° for PU to 90.0° for PU whit 20 wt.% of lignin
Polypropylene (PP) [69]	Kraft lignin acid (5.0 wt.%)	Adhesion properties	107.2° for PP and 92.46° for PP whit Kraft lignin (acid)
Polystyrene (PS) [70]	Alkali lignin (6.5 wt.%) modified whit trimethyl quaternary ammonium salt	Antibacterial activity	55.9° for PS and 10.5° for PS whit lignin
